# The Mediating Role of Parental Emotional Distress in the Relationship Between Neuroticism and Children’s Emotional and Behavioral Problems: A Network Analysis and Structural Equation Modeling Study

**DOI:** 10.3390/bs16071135

**Published:** 2026-07-06

**Authors:** Min Xie, Yaqing Huang, Lan Wen, Haiyan Cui, Shuyue Zhang

**Affiliations:** 1 Faculty of Education, Guangxi Normal University, Guilin 541004, China; xm1997@stu.gxnu.edu.cn (M.X.); yqh1998@stu.gxnu.edu.cn (Y.H.); chy@stu.gxnu.edu.cn (H.C.); 2 South China Business College, Guangdong University of Foreign Studies, Guangzhou 510545, China; 204043@gwng.edu.cn

**Keywords:** emotional and behavioral problems, neuroticism, emotional distress, structural equation modeling, network analysis

## Abstract

Emotional and behavioral problems (EBPs) experienced by children have become an important global health issue requiring immediate attention. Previous studies have shown that parental neuroticism is associated with EBPs in children. However, the mechanisms underlying this association have yet to be fully elucidated. This study examined the relationship between parental neuroticism and preschoolers’ EBPs, focusing on the mediating role of parental emotional distress (e.g., anxiety, depression, and somatization). In addition, to gain a deeper understanding of children’s emotional and behavioral difficulties, this study constructed a comprehensive network of preschoolers’ EBPs to investigate the interconnections among individual symptoms. A total of 1216 Chinese families (M_children age_ = 4.46 years; 47.6% girls) participated in this study, completing the Chinese Big Five Personality Inventory, Brief Symptom Inventory, and Strengths and Difficulties Questionnaire. Data analysis was conducted using structural equation modeling (SEM) and network analysis. The results showed that parental neuroticism was positively associated with children’s EBPs, and this relationship was partially mediated by parental emotional distress. “Constantly fidgeting or squirming”, “stealing from home, kindergarten, or other places”, “often unhappy‚ depressed or tearful”, and “many fears‚ easily scared” emerged as the most central symptoms in the network of EBPs. These findings hold significant implications for enhancing well-being among parents and their preschool children, suggesting that parents should prevent the spread of negative emotions such as anxiety and depression. Timely, targeted interventions focusing on central symptoms of EBPs are essential for promoting children’s mental well-being.

## 1. Introduction

Early childhood is a vital period for social, emotional, and behavioral development. During this period, the places where children live and interact expand from the family to various venues such as kindergartens and communities. Preschool children may begin to show signs of emotional and behavioral problems (EBPs), which comprise emotional difficulties (e.g., anxiety, depression, loneliness, rumination, and somatic complaints; Madigan et al., 2013) and behavioral difficulties (e.g., aggression, attention problems, defiance, and peer problems; Perkinson-Gloor et al., 2015; Yu, 2022). Previous empirical research has demonstrated that emotional and behavioral difficulties frequently co-occur and are positively correlated with each other (Waszczuk et al., 2021; Xie et al., 2024). In recent decades, studies across various cultures and countries have reported an increase in the prevalence of these problems, indicating a growing global concern about the mental health of children (Idris et al., 2019; Kiros et al., 2025). In China, recent studies conducted in the past two years have reported that the prevalence of emotional and behavioral problems in children ranged from 5.5% to 23.83% (Cao et al., 2024; Hou et al., 2026). Existing empirical studies have consistently shown that EBPs have significant negative effects on children’s cognitive, social, and academic development (Liu et al., 2022). If left unaddressed, EBPs may persist into adolescence and adulthood, leading to long-term consequences such as neurodevelopmental disorders (Wang et al., 2025), emotional instability (Ran et al., 2021), asocial behavior (M. Yang et al., 2022), poor academic performance, social exclusion, and suicidality (Chen et al., 2023). Network analysis has been widely used in understanding the comorbidity of mental health problems, as well as providing hints for people to prevent and treat comorbidities by identifying central symptoms that are most influential to other symptoms (Jones, 2020). Researchers have explored the characteristics of the emotional and behavioral problems network among adolescents (e.g., Speyer et al., 2022; Xie et al., 2024). However, up to now, there have been few studies using network analysis to examine the network structure of a broad range of emotional and behavioral problems in preschool children in China. Thus, the present study aims to examine the network structure of EBPs and identify the central symptoms.

In addition, given the disturbing consequences of EBPs, factors involved in the occurrence of EBPs should be examined to produce suitable, effective treatment plans. A growing body of prospective evidence indicates that various parental factors are associated with the occurrence and aggravation of preschoolers’ EBPs (Chen et al., 2023; Madigan et al., 2013; Prinzie et al., 2005). Parents play a substantial and unique role in children’s emotional and behavioral development, and generally, examined risk factors for children’s EBPs are parental personality traits. Neuroticism, as an important personality trait, is characterized by emotional instability, negative affect, and a lack of self-awareness (Lone & Albotuaiba, 2022). According to the Bioecological Model of Human Development (Bronfenbrenner & Morris, 2006), parental neuroticism constitutes a person’s “force characteristics,” which are theorized to be associated with parent–child interactions and related to the emotional and behavioral development of children. Thus, the present study also aims to examine the direct and indirect pathways linking parental neuroticism to preschool children’s EBPs within a Chinese sample.

### 1.1. Parental Neuroticism and Children’s Emotional and Behavioral Problems

The Five-Factor Model (FFM) of personality (i.e., neuroticism, conscientiousness, extraversion, agreeableness, and openness) has generated substantial interest among personality researchers (McCrae & Costa, 2008). Compared with other personality traits, neuroticism is more directly indicative of a person’s ability to regulate emotions. It describes the stability of an individual’s emotions, the degree of inner distress, and the tendency towards depression. Parents with a high degree of neuroticism usually exhibit characteristics such as sensitivity, suspicion, anxiety, depression, vulnerability, irritability, and the tendency to lose their temper. On the contrary, parents with a low degree of neuroticism tend to be resilient, confident, composed, and calm.

Evidence shows that parental neuroticism has been linked to children’s defiance, anger, antisocial behavior, delinquency, and more generally, to internalizing and externalizing problems in children of any age group (Prinzie et al., 2004; Han et al., 2010). A longitudinal study has shown that parental neuroticism has a consistent positive direct association with emotional and behavioral problems in children from toddlerhood through elementary school ages (Oliver et al., 2009). According to Belsky’s (1984) process model of parenting, the determinants of the parenting process come from multiple domains, including parent characteristics (such as personality characteristics and psychological functioning), child characteristics (such as temperament), and social contexts (such as work habits, social support, and marital relationships). Belsky emphasized the significance of personality in the domain of parent characteristics, arguing that it plays a crucial role as the primary determinant of children’s emotional and behavioral development. However, there are conflicting viewpoints regarding how the neuroticism of parents affects the emotional and behavioral development of their children (Yan et al., 2024). One viewpoint proposes that parental personality traits may be directly associated with children’s emotional and behavioral development through mechanisms such as genetic factors (Prinzie et al., 2005). Parental neuroticism can directly increase the probability of emotional and behavioral problems in their offspring through genetic inheritance (Ask et al., 2021). Another viewpoint suggests that a high level of neuroticism in parents is consistently linked with poor psychosocial functioning, such as anxiety, depression, and stress (Ellenbogen & Hodgins, 2004). These negative emotions, in turn, are associated with more emotional and behavioral problems in children. Given the complex nature of the relationship between parents’ neuroticism and children’s emotional and behavioral development, this study aims to examine whether parental neuroticism impacts preschool children’s emotional and behavioral problems directly or indirectly by considering potential mediating variables related to psychosocial functioning, such as emotional distress (e.g., anxiety, depression, and somatization). Thus, this study proposes Hypothesis 1: Parental neuroticism is positively associated with emotional and behavioral problems in preschool children.

### 1.2. Emotional Distress as a Mediator

Emotional distress (e.g., anxiety, depression, and somatization) is a common mental health problem in parents. Studies consistently reported higher levels of emotional distress in parents (Aktar et al., 2017; Wang et al., 2023; W. Yang et al., 2021). More specifically, depressive symptoms are prevalent among mothers of young children and affect approximately 19.9% of mothers during the postnatal period (Cena et al., 2021). The prevalence of maternal anxiety varies depending on which disorders are included in the estimate, but can be as high as 13.0% (Munn et al., 2014). Consequently, large numbers of children are exposed to parental anxiety and depression. Empirical evidence corroborates relations between parental personality traits and emotional distress. For example, numerous studies have established that among the Big Five personality traits, neuroticism is the strongest predictor of depression-proneness (Goodwin & Gotlib, 2004), depressive symptoms (Chioqueta & Stiles, 2005; Karreman et al., 2013), and clinical depression (Kotov et al., 2010). Higher levels of neuroticism increased the odds of psychiatric comorbidity among individuals with anxiety and depressive disorders (Nolen-Hoeksema et al., 1999). However, existing studies have focused more on clinical parents; their findings may not be generalizable to the general population, as patients with clinical depression or anxiety disorders differ from non-clinical individuals.

Emotional contagion has long been regarded as the automatic transfer of emotional states between individuals. The emotional contagion theory states that individuals automatically imitate the facial, vocal, and postural nonverbal cues of emotions of others and, through a process of afferent feedback, converge emotionally (Hatfield et al., 1993). This phenomenon has been observed in young children. According to this theory, parental negative emotions can spread like an infectious disease in social networks and may consequently promote interpersonal emotional convergence (Hatfield et al., 1993). Thus, over time, parental emotional distress eventually should threaten the health and well-being of preschool children living in the home. Previous observational studies have demonstrated an association between parental depression or anxiety and increased risk of EBPs in early childhood (Barker et al., 2011). Children of parents who display symptoms of anxiety and/or depression show greater externalizing (e.g., aggression, overactivity) and internalizing (e.g., shyness, withdrawal) symptoms in early childhood (S. H. Goodman et al., 2011). Thus, this study proposes Hypothesis 2: Parental emotional distress mediates the relationship between neuroticism and children’s emotional and behavioral problems.

### 1.3. Current Study

The aims of the present study are threefold. First, we aim to examine the direct link between parental neuroticism and preschool children’s EBPs. Based on Belsky’s process model of parenting and previous literature, we hypothesized that parental neuroticism is positively associated with emotional and behavioral problems in preschool children (Hypothesis 1). Second, we aim to use latent variable structural equation modeling (SEM) to establish the relationships among parental neuroticism, emotional distress, and children’s EBPs. Previous research has found that parental neuroticism is related to increased children’s EBPs. Yet little research has examined affective process variables that may account for this relationship. Drawing on emotional contagion theory and related research, this study developed and tested a mediation model to examine the mediating role of emotional distress. We hypothesized that parental emotional distress mediates the relationship between neuroticism and children’s emotional and behavioral problems (Hypothesis 2). Third, we aim to construct network models of emotional and behavioral problems based on symptoms through network analysis. Under the latent variable perspective, higher-order constructs (e.g., the ‘p’ factor) are used to represent various manifestations of psychopathology in children, while possible local associations between specific symptoms (e.g., constantly fidgeting or squirming) may be ignored (Borsboom, 2017). Therefore, in order to gain a deeper understanding of the emotional and behavioral problems of preschool children, the study adopted the network analysis method to explore the local correlations among individual symptoms and identify the central symptoms that have the greatest impact on other symptoms. In this study, network analysis is a descriptive method that provides graphical representations of patterns in correlation matrices, without making any assumptions. In conclusion, to the best of our knowledge, this is the first study to investigate the symptom-level interconnectedness of emotional and behavioral problems in Chinese preschool children via network analysis, and to explore the mediating pathway from parental neuroticism to children’s EBPs through parental emotional distress.

## 2. Materials and Methods

### 2.1. Participants and Procedure

The study employed a convenience sampling method, targeting children and their parents from five kindergartens in Shenzhen and Guangzhou, Guangdong Province, China. After we completed the questionnaire compilation on the SoJump platform, we recruited one principal from each kindergarten to serve as the survey administrator. The informed consent form and questionnaires were sent to the preschoolers’ parents by the principal of the kindergarten via WeChat and were completed online. Participants were guaranteed full confidentiality and anonymity, and were explicitly informed that their involvement was voluntary, with the right to withdraw at any time without penalty. The survey and research procedures were approved by the ethics committee of the first author’s university.

A total of 1232 parents and their children participated in this survey, and sixteen participants were removed because they did not follow the instructions (e.g., regular responses like selecting ‘1’ for all items, or failing to choose ‘strongly agree’ on attention-check items). In total, 1216 children (M_age_ = 4.46 years, SD_age_ = 0.87) and their parents (M_age_ = 35.65 years, SD_age_ = 4.65; see Table 1 for more details) were included in the sample, yielding an effective response rate of 98.70%. This high response rate indicates a strong level of engagement from the participants, enhancing the reliability of the findings. Of the 1216 participants, 30 cases (2.5%) had missing data on the parental age variable, while all other observed indicator variables had complete data with no missing values. To handle this minimal missingness, we employed Full Information Maximum Likelihood (FIML) estimation with robust standard errors (MLR). Among the participating primary caregivers, 978 (80.43%) were mothers, while the remaining 238 (19.67%) were fathers. This gender distribution is consistent with the prevailing familial division of childcare responsibilities in contemporary Chinese society, where mothers typically serve as the primary informants regarding children’s daily routines and educational activities. The sample of children consisted of 579 girls (47.62%) and 637 boys (52.38%), with a balanced gender ratio.

### 2.2. Instruments

Neuroticism. The Chinese Big Five Personality Inventory-15 (CBF-PI-15; Zhang et al., 2019) is a validated brief measure of the Big Five personality traits in Chinese populations, with each dimension comprising three representative items. It comprises fifteen self-reported items, with responses ranging from 1 (disagree strongly) to 6 (agree strongly). We used the neuroticism subscale to evaluate parental neuroticism. Confirmatory factor analysis (CFA) supported that the three items converge on a common factor (CFI = 1.000, TLI = 1.000, RMSEA = 0.000, SRMR = 0.000). In the current sample, Cronbach’s alpha for the neuroticism subscale was 0.86, indicating acceptable internal consistency.

Emotional distress. The Brief Symptom Inventory (BSI–18; Li et al., 2018) was used to assess parental emotional distress. The inventory consists of three symptom dimensions of anxiety, depression, and somatization, each comprising six items. Responses were rated on a 5-point Likert-type scale, ranging from 1 (not at all) to 5 (very much). This scale demonstrated acceptable model fit in CFA (CFI = 0.915, TLI = 0.901, RMSEA = 0.052, SRMR = 0.046), supporting the validity of the construct. In the current sample, Cronbach’s alphas for the full scale, anxiety, somatization, and depression subscale items were 0.89, 0.96, 0.91, and 0.93, respectively, indicating good internal consistency.

Emotional and behavioral problems. The parent-reported version of the Strengths and Difficulties Questionnaire (SDQ; R. Goodman, 1997) was utilized to identify preschool children’s emotional and behavioral problems. The SDQ includes 25 items, five subscales of five items each: hyperactivity–inattention, emotional symptoms, peer relationships, conduct problems, and prosocial behaviors. Responses were rated on a 3-point Likert-type scale, ranging from 0 (not true) to 2 (certainly true). Items 7, 11, 14, 21, and 25 are scored in reverse order. The first four subscales are summed to yield a total EBPs score. This score has standardized cutoffs based on population norms, categorizing results into abnormal (10%), borderline (10%), and normal (80%) ranges (S. H. Goodman et al., 2011). This scale demonstrated acceptable model fit in CFA (CFI = 0.901, TLI = 0.876, RMSEA = 0.051, SRMR = 0.064), supporting the validity of the construct. In the current sample, Cronbach’s alphas for the full scale, hyperactivity–inattention, emotional symptoms, peer relationships, and conduct problems subscale items were 0.81, 0.70, 0.71, 0.72, and 0.72, respectively, indicating acceptable internal consistency.

Demographic data. Parents reported family and child demographic information, such as family monthly income, their gender, age, educational attainment, and their child’s gender, age, and class. Socioeconomic status (SES) was operationalized as a composite score of family monthly income and parental educational attainment.

### 2.3. Statistical Analysis

Descriptive statistics were calculated using SPSS 26. A heatmap of the Pearson correlation matrix was generated using Python 3.12. CFA and SEM were conducted using Mplus 8.0 (Muthen & Muthen, 1998–2017). Network analysis was conducted using the *qgraph* (version 1.6.5; Epskamp et al., 2012), *bootnet* (version 1.4.3; Epskamp et al., 2018), *mgm* (version 1.2; Haslbeck & Waldorp, 2020), and *networktools* (version 1.2; Jones, 2020) packages in R (version 4.1.0; R Core Team, 2021).

CFA and SEM. CFA was conducted to evaluate the measurement model. Given potential non-normality, the robust maximum likelihood estimator (MLR) was used to provide robust standard errors and fit statistics. Model fit was evaluated using the Comparative Fit Index (CFI), Tucker Lewis Index (TLI), Root Mean Square Error of Approximation (RMSEA), and Standardized Root Mean Square Residual (SRMR). According to common fit criteria (Hu & Bentler, 1999), if the values of the CFI and TLI were greater than 0.90 and the values of the RMSEA and SRMR were less than 0.08, then the models were deemed to be well fitting. Following Kline’s (2015) recommendation, we evaluated the model using multiple indicators and applied the majority rule when certain indicators failed to meet the criteria. Then, to verify our hypothesis model, SEM was employed to examine the direct and indirect paths between parental neuroticism and children’s emotional and behavioral problems. We used the same estimation approach for SEM as for CFA. We also evaluated the significance of the statistical mediation using an accelerated bias-corrected bootstrapping analysis with 5000 bootstrap samples. According to common fit criteria (MacKinnon et al., 2004), if the 95% confidence interval (CI) excluded 0, the estimation was deemed significant.

Network analysis. To achieve a more in-depth understanding of children’s EBPs, we constructed a comprehensive network of emotional and behavioral problems at the symptom level. The network was regularized based on the graphical LASSO (least absolute shrinkage and selection operator) with EBIC (extended Bayesian information criterion model selection; r = 0.5) to shrink small edges to exactly zero, in order to create a sparse network that is easier to visualize and interpret (Epskamp et al., 2012). In the network analysis, each item was defined as a node, and each pairwise association between items was defined as an edge.

To gain insight into the relative importance of each node, expected influence (EI) was estimated via the R-package *qgraph*. Nodes having higher EI were considered to be more important in the network. Additionally, predictability, which reflects how well a given node is predicted by all its surrounding nodes (Haslbeck & Fried, 2017), was calculated via the R-package *mgm*. The value of the predictability of each node was added to the network as a pie chart in the outer ring of the node, and the mean predictability of each domain was calculated to aid the interpretation of the network.

To assess the robustness of the observed network model, the accuracy and stability of the network were assessed using the R-package *bootnet* based on 1000 bootstraps performed for each node. Firstly, the accuracy of edge-weights was estimated through non-parametric bootstrap techniques. Secondly, the stability of the network was assessed by a case-dropping bootstrap procedure. As recommended previously (Epskamp et al., 2018), a correlation stability coefficient (CS) should not be lower than 0.25 and is preferably above 0.5; values greater than 0.25 indicate moderate stability, while values greater than 0.5 indicate strong stability. Finally, differences in network properties (i.e., edge-weights, node-EIs) were evaluated by bootstrapped difference tests (Epskamp et al., 2018).

## 3. Results

### 3.1. Common Method Bias (CMB) Assessment and Correlations

**Figure 1:**
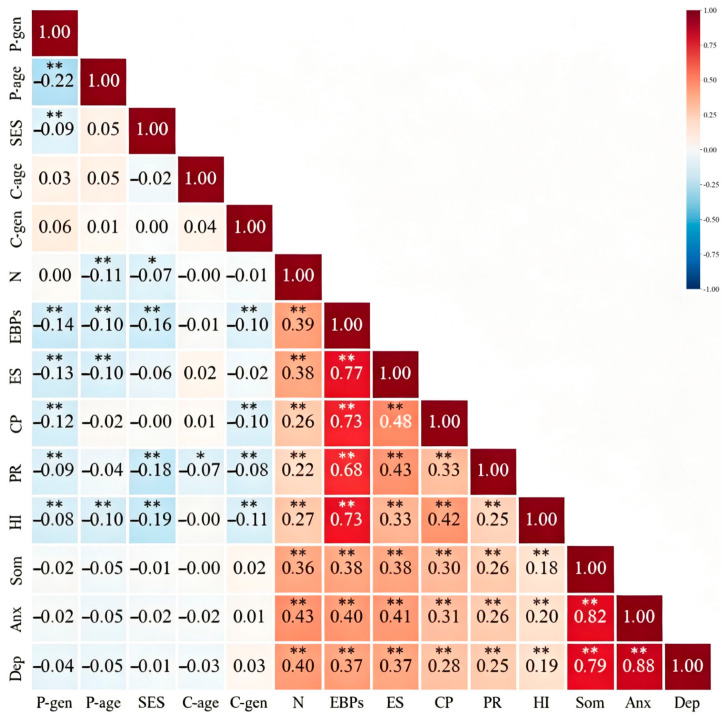
Heatmap of the Pearson correlation matrix. Note: The number in each box is the Pearson correlation coefficient. P-gen = Parental gender; P-age = Parental age; C-gen = Children’s gender; C-age = Children’s age; N = Neuroticism; EBPs = emotional and behavioral problems; ES = Emotional symptoms; CP = Conduct problems; PR = Peer relationships; HI = Hyperactive-inattention; Anx = Anxiety; Dep = Depression; Som = Somatization; SES = Socioeconomic status. (* *p* < 0.05, ** *p* < 0.01).

To assess the potential impact of CMB, Harman’s single-factor test was conducted. The results showed that there were 11 factors with eigenvalues greater than 1, and the first factor could explain 27.90% of the variability in the data, which was less than the critical threshold of 40% (Podsakoff et al., 2003). While this suggests that CMB is unlikely to severely distort the results, we acknowledge that this statistical procedure has limited sensitivity and cannot completely rule out residual common method or informant biases. Therefore, we interpret the findings with caution regarding potential shared method variance. Pearson’s correlations (Figure 1) suggested that the neuroticism of parents was significantly positively correlated with preschool children’s emotional symptoms (r = 0.38), hyperactive-inattention (r = 0.27), conduct problems (r = 0.26), peer relationships (r = 0.22), and total EBPs score (r = 0.39). Additionally, parental emotional distress was significantly positively correlated with neuroticism and with preschool children’s total EBPs score. More specifically, parental anxiety was significantly positively correlated with neuroticism (r = 0.43) and with preschool children’s total EBPs score (r = 0.40). Parental depression was significantly positively correlated with neuroticism (r = 0.40) and with preschool children’s total EBPs score (r = 0.37). Parental somatization was significantly positively correlated with neuroticism (r = 0.36) and with preschool children’s total EBPs score (r = 0.38).

### 3.2. Mediation Analysis: Relation Between Parental Neuroticism, Emotional Distress, and Children’s EBPs

Before testing the mediation model, CFA was conducted to evaluate the measurement model. To improve model efficiency, item parceling was applied. Following the recommendations of Matsunaga (2008), we used the internal consistency method to construct latent variables for children’s emotional and behavioral problems comprising emotional symptoms (skewness = 1.555, kurtosis = 3.100), conduct problems (skewness = 1.181, kurtosis = 2.212), hyperactive–inattention (skewness = 0.559, kurtosis = 0.033), and peer relationships (skewness = 0.398, kurtosis = −0.162) as indicators and emotional distress comprising depression (skewness = 3.088, kurtosis = 11.864), anxiety (skewness = 3.211, kurtosis = 12.649), and somatization (skewness = 3.759, kurtosis = 18.440) as indicators. Parental neuroticism was represented by tree indicators: neuroticism1 (skewness = 0.121, kurtosis = −0.606), neuroticism2 (skewness = 0.161, kurtosis = −0.696), and neuroticism3 (skewness = 0.307, kurtosis = −0.684). Given the non-normal distribution of the emotional distress scores, the measurement model was estimated using the MLR estimator. The results indicated that the measurement model fit the data well, *χ*^2^ = 93.793, *df* = 32, *p* < 0.001, CFI = 0.987, TLI= 0.982, RMSEA = 0.040 (90% CI = [0.031, 0.049]), SRMR = 0.027. Significant standardized loadings were found for all indicators on their respective factors (see Table 2). The factor loading for the peer relationships was relatively low (λ = 0.496), marginally below the conventional threshold (0.500). This might reflect the broader content of this SDQ subscale, which encompasses both the positive and negative aspects of interactions with peers.

**Figure 2:**
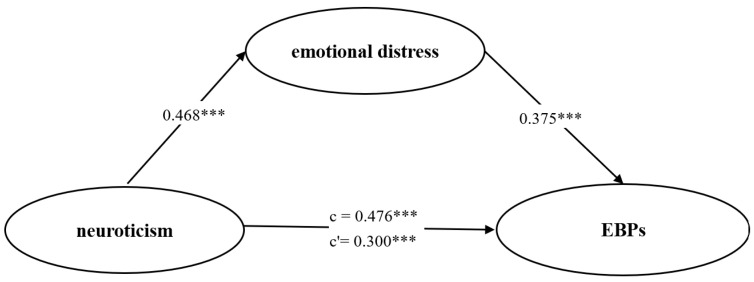
Mediation model between neuroticism, emotional distress, and children’s EBPs. (*** *p* < 0.001).

Direct and indirect pathways between parental neuroticism and children’s EBPs were tested using SEM. We analyzed a mediation model (Figure 2), with parental neuroticism as the independent variable, children’s EBPs as the outcome variable, emotional distress as the mediator, and parental and children’s age and gender, as well as SES, as covariates. The statistical mediation of emotional distress was tested using the bias-corrected non-parametric percentile bootstrap method. Mediation model fit the data well, *χ*^2^ = 222.106, *df* = 59, *p* < 0.001, CFI = 0.976, TLI= 0.969, RMSEA = 0.048 (90% CI = [0.041, 0.054]), and SRMR = 0.035, with factor loadings ranging from 0.671 to 0.886 (all *p*s < 0.001). This model explained 38.7% variance in EBPs and 21.9% variance in emotional distress. The estimated total effect of parental neuroticism on EBPs, as derived from the cross-sectional data, was significant (B = 0.476, SE = 0.033, 95% CI = [0.410, 0.539]). Parental neuroticism was positively associated with emotional distress (B = 0.468, SE = 0.024, *p* < 0.001). Emotional distress was positively associated with children’s EBPs (B = 0.375, SE = 0.056, *p* < 0.001). The direct pathway linking parental neuroticism to children’s EBPs was significant (B = 0.300, SE = 0.043, 95% CI = [0.218, 0.384]). Additionally, the indirect pathway from parental neuroticism to children’s EBPs via emotional distress was significant (B = 0.175, SE = 0.028, 95% CI = [0.119, 0.233]). Therefore, parental emotional distress played a mediating role between parental neuroticism and children’s emotional and behavioral problems. H1 and H2 were supported.

### 3.3. Network Analysis: Network Structure of EBPs

**Figure 3:**
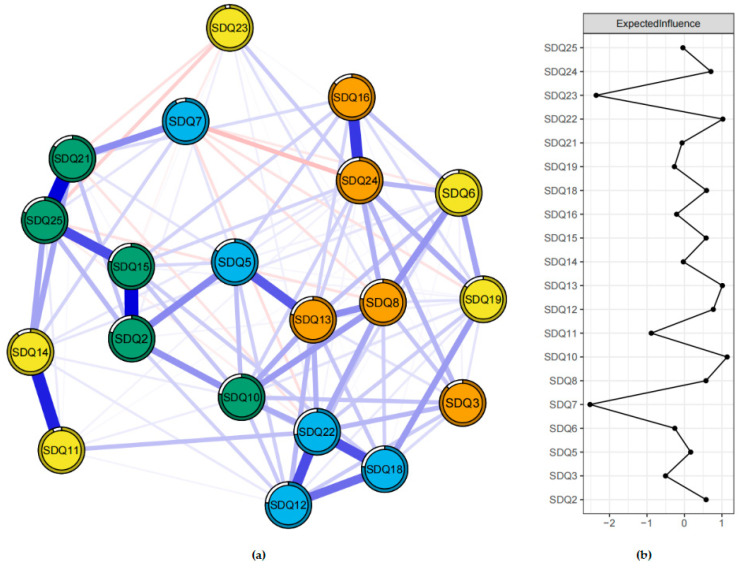
Results of network analysis. (**a**) Network model of EBPs; (**b**) Centrality plots for the 20 symptoms depicted as Expected Influence of EBPs symptoms. Note: Blue lines are positive connections and red lines are negative connections. The thickness of the line represents the connection strength. Thicker edges indicate higher correlations. Colored areas in the rings surrounding the nodes represent the node predictability (percentage of variance of a given node explained by surrounding nodes). The blue nodes belong to the “conduct problems” dimension; the green nodes belong to the “hyperactive-inattention” dimension; the yellow nodes belong to the “peer relationships” dimension; and the orange nodes belong to the “emotional symptoms” dimension.

Network pattern. Figure 3 illustrates the estimated network structure of the children’s emotional and behavioral problems based on the symptom level, including 20 nodes. Regarding basic characteristics of the network, first, 113 of the 190 possible edges (59.5%) were not zero and reflected considerable interconnectedness between symptoms (M_weight_ = 0.037). The edge between SDQ21 (Thinking before doing) and SDQ25 (Good attention) was strongest (r = 0.33), followed by edges SDQ15–SDQ2 (Easily distracted‚ concentration wanders–Restless‚ overactive‚ cannot stay still for long; r = 0.32), SDQ11–SDQ14 (Have good friends–Generally liked by peers; r = 0.28), SDQ16–SDQ24 (Nervous or clingy in new situations–Many fears‚ easily scared; r = 0.25). Edge values of the network can be found in Supplementary Information (Table S1). Bootstrapped edge weight differences suggested that the strongest edge weights were reliably stronger than most other edge weights (Figure S3a).

Predictability and EI. The mean node predictability for the EBPs symptoms network was 30.0%. This meant that, on average, 30.0% of the variance of each node was explained by its neighbors in the network. The highest predictability scores were observed for SDQ22 (Stealing from home, kindergarten, or other places; predictability = 46.6%), SDQ18 (Often lies or cheats; predictability = 41.2%), and SDQ12 (Often fights with other children or bullies them; predictability = 41.1%). The predictability of SDQ23 (Gets along better with adults than with other children; predictability = 6.7%) was the lowest. In terms of domains, hyperactivity–inattention had the highest mean predictability (35.6%), and peer relationships had the lowest mean predictability (17.8%). The results of EI are shown in Figure 3b. The most central nodes, according to the EI index, were SDQ10 (Constantly fidgeting or squirming; EI = 1.14), SDQ22 (Stealing from home, kindergarten, or other places; EI = 1.03), SDQ13 (Often unhappy‚ depressed or tearful; EI = 1.01), and SDQ24 (Many fears‚ easily scared; EI = 0.71), suggesting that these individual symptoms were the most influential within the entire symptom network model in terms of variance explained. On the contrary, other symptoms such as SDQ7 (Generally well behaved‚ usually does what adults request) and SDQ23 (Gets along better with adults than with other children) had a marginal impact in this network. The bootstrapped differences test showed that the higher centrality indices were statistically different from the lowest (Figure S3b).

Within-network accuracy and stability. In regard to the robustness of the EBPs symptoms network, firstly, the bootstrapping stability test of edge-weights showed that the constructed network was stable (Figure S1). Then, EI values indicated a good level of stability (CS equaled 0.75), which was greater than the recommended value of 0.5, suggesting that 75% of participants could be dropped from analyses without significantly changing the network structure (Figure S2).

## 4. Discussion

Belsky’s process model of parenting and prior literature suggest that parental neuroticism plays a significant role in preschool children’s emotional and behavioral development; however, there is insufficient research on the statistical mediating mechanisms underlying the relationship between neuroticism and emotional and behavioral problems (EBPs). Drawing on Belsky’s process model of parenting, emotional contagion theory, and related research, this study discovered emotional distress as a mediating variable in the association between parental neuroticism and children’s EBPs. In order to gain a deeper understanding of the EBPs of preschool children, this study also examined the network structure of EBPs from a network perspective and identified the central symptoms. Our findings offer some directions for the intervention to lessen EBPs among preschool children. The integration of SEM and network analysis in this study offers complementary insights into the relationships among parental neuroticism, emotional distress, and children’s EBPs, following the analytic framework of previous research (e.g., Luo et al., 2025; Zeng et al., 2025). SEM provided a confirmatory, theory-driven test of the hypothesized mediating pathways at the latent construct level, examining how parental neuroticism relates to children’s EBPs through emotional distress. Network analysis, in contrast, adopted an exploratory, data-driven approach to uncover the internal structure of EBPs’ symptoms at the item level, identifying which specific symptoms were most central within the symptom network. While both methods highlighted the prominence of emotional symptoms, they do so from distinct methodological angles: SEM identifies the relative strength of associations linking parental psychological characteristics to child outcomes within a theoretical mediation framework, whereas network analysis reveals the structural centrality and local connectivity of specific symptoms within the child symptom network. Thus, the two methods are not equivalent; rather, they are complementary. This methodological complementarity strengthens our conclusions by demonstrating that emotional symptoms are robustly implicated across both macro-level associational models and micro-level symptom networks, offering a more nuanced and multifaceted understanding.

### 4.1. Characteristics of Emotional and Behavioral Problems in Preschool Children

Among the four dimensions of emotional and behavioral problems, hyperactive-inattention had the highest score, indicating that hyperactive-inattention among preschool children was the most severe. Attention-deficit hyperactivity disorder (ADHD) has become a common and major global public concern, including in China. A few studies have reported that approximately 23% of the children suffer from subthreshold ADHD symptoms (Balazs & Kereszteny, 2014). Tong et al. (2013) reported that the number of individuals diagnosed with ADHD has increased, and the pooled national prevalence is 5.7% in China. While ADHD symptoms must have an age of onset before age 12 to warrant a diagnosis of ADHD, ADHD symptoms such as hyperactivity and inattention usually first appear before age 6 (Blackman, 2016). In order to prevent the formation and deterioration of ADHD, parents should promptly intervene in the “constantly fidgeting or squirming” symptom of preschool children, as the network analysis has found that it is the core symptom of the hyperactive-inattention domain.

Childhood emotional and behavioral problems are unlikely to be transient and, especially if they remain stable, can impair the children’s functioning and development. In the network of the symptoms of EBPs, “constantly fidgeting or squirming”, “stealing from home, kindergarten, or other places”, “often unhappy‚ depressed, or tearful”, and “many fears‚ easily scared” were identified as the most central symptoms, as indicated by EI. Targeted interventions for these symptoms are important to alleviate the overall level of emotional and behavioral difficulties in this population. Among the four central symptoms, two of them (i.e., “often unhappy‚ depressed or tearful” and “many fears‚ easily scared”) were from the domain of emotional symptoms. The results were in keeping with the existing evidence that “emotional symptoms” acted as a central domain in the network of EBPs in Chinese samples (Xie et al., 2024). Preschool children have a low level of emotional regulation (Song et al., 2024). When children encounter difficulties in their daily lives, they often have difficulty regulating their emotions. Consequently, they express their inner dissatisfaction through external manifestations such as crying.

### 4.2. Parental Neuroticism Is Associated with EBPs in Preschool Children

The significant association between parental neuroticism and EBPs in children is consistent with previous studies (Ellenbogen et al., 2010; Mazza et al., 2020). For instance, a cross-sectional study conducted in China has shown that children of parents with high levels of neuroticism have higher EBPs scores (Zhang & Li, 2016). Similarly, a longitudinal study has revealed that neuroticism in parents, assessed when the children were between four and 12 years of age, predicted poor peer relationships and interpersonal functioning in children 10 years later as they transitioned from late adolescence to early adulthood (Ellenbogen et al., 2010). In contrast, another longitudinal study has demonstrated that emotional stability in parents, assessed when the offspring were between 4 and 7 years of age, was negatively associated with externalizing problem behaviors in offspring (Prinzie et al., 2005). Building on this evidence, this research further explored the extensive associations between parental neuroticism and various emotional and behavioral problems (i.e., hyperactive-inattention, emotional symptoms, peer relationships, and conduct problems) in preschool children.

Our findings are also consistent with social learning theory, which supports the argument that learning through the observation and imitation of parents is crucial for children’s acquisition of, or change in, emotions and behaviors. Parents with high scores on neuroticism tend to be easily distressed, anxious, tense, nervous, and have difficulties in coping with daily stressors (Prinzie et al., 2009). On the one hand, preschool children with the same inherited personality traits have a propensity to engage in more hostile interactions and are at risk of developing emotional problems (Prinzie et al., 2005). On the other hand, emotional problems in children may be an imitation of these explosive reactions (Brook et al., 2002). Parents are the primary emotion socializing agents for preschool children, and may socialize children’s emotions through: (1) reactions to children’s emotions, (2) emotion-related conversations, and (3) emotional expressiveness in relationships (Tobin et al., 2000). Higher levels of neuroticism were associated with lower typically adaptive emotion regulation strategies (reappraisal, acceptance, problem solving, and mindfulness) and greater typically maladaptive emotion regulation strategies (avoidance and suppression) (Baranczuk, 2019). The non-adaptive emotional regulation strategies adopted by parents with high neuroticism are not conducive to the emotional socialization of young children. In addition, parental neuroticism is associated with inconsistent, hostile, and confrontational interactions, which may contribute to behavioral problems in children, including defiance, aggression, antisocial tendencies, and withdrawal (Prinzie et al., 2009). It is manifested in prior studies that children with EBPs would be expected to create unusual stress on parents, so that a neurotic parent may more often experience a failure in parenting effectiveness, resulting in further escalation of emotional problems (Prinzie et al., 2005).

### 4.3. The Mediating Role of Emotional Distress

In this cross-sectional mediation analysis, parental emotional distress emerged as a statistical mediator in the relationship between parental neuroticism and children’s EBPs. Specifically, a higher level of neuroticism was associated with greater emotional distress (e.g., anxiety, depression, and somatization), which is a well-established correlate of emotional and behavioral problems in preschool children. This finding is consistent with emotional contagion theory, which proposes that parental neuroticism is associated with offspring behavior via the transfer of negative emotions such as anxiety and stress. According to emotional contagion theory, preschool children mainly capture and synchronize their parents’ emotions through two stages: mimicry and feedback (Hatfield et al., 1993). Distressed parents lack good emotional regulation skills and thus are unable to provide their offspring with models of regulated functioning (Campbell et al., 2000). Especially when children are highly sensitive to their parents’ negative emotional states, they may synchronize their movements with parental emotional displays with the function of facilitating interactions. After mimicking the negative emotions of their parents, preschool children actually experience an emotional state that is consistent with the information that was sent through the process of afferent feedback, that is, the process by which information from the peripheral nerves is sent to the brain (Rueff-Lopes et al., 2015). As a result, children of distressed parents may adopt maladaptive coping strategies as a defense against overwhelming negative emotions, which could manifest as various internalizing and externalizing problems (Wadsworth, 2015). In addition, emotional distress in parents may create emotionally turbulent environments within the family, exposing children to chronic stress (Guidetti et al., 2016). Chronic stress may further cause a cascade of abnormal physiological and mental changes in preschool children. For instance, it is associated with structural alterations in key brain areas responsible for emotional regulation and executive functions (El-Sheikh & Erath, 2011), thereby increasing the risks of both emotional (e.g., depression, anxiety, and rumination) and behavioral problems (e.g., hyperactivity–inattention, and conduct problems) in preschool children. From the perspective of upbringing, the parental role requires concern for others, and parents with greater ability to empathize with the child are probably better able to identify and respond to children’s needs (Prinzie et al., 2009), as well as tend to have more positive attributions regarding the children’s behavior. However, a disposition to experience emotional distress might limit parents’ ability and willingness to respond adequately to children’s signals. Evidence suggests that distressed parents tend to be less responsive, less engaged, and adopt strict but ineffective parenting styles, all of which are associated with the formation of an insecure attachment relationship between parents and children (Campbell et al., 2000).

### 4.4. Implications and Limitations

There are important theoretical and practical implications for these findings. From a theoretical point of view, most of our knowledge about personality traits and children’s outcomes comes from research on parenting style, particularly warmth, behavioral control, and autonomy support (Prinzie et al., 2009). This study expands our knowledge of how parental neuroticism is associated with child outcomes through psychological mediators in a cross-sectional sample. That is, emotional distress plays a mediating role in the relationship between parents’ neuroticism and EBPs of preschool children. This research also enabled the most important symptoms in the structure of emotional and behavioral problems to be identified and, therefore, assisted in elucidating the specific symptoms of greatest importance to the emotional well-being of preschool children. In practical terms, our results can shed light on preventative and intervention strategies aimed at EBPs of preschool children. Given that parents’ personality is stable over time (Terracciano et al., 2006), intervention and prevention programs that target emotional distress and children’s emotional and behavioral problems may yield better developmental outcomes for children’s emotional and behavioral development.

There are some limitations to the current study that should be mentioned. First, parents reported on all variables, which may introduce common method bias and social desirability bias. Although the results of the Harman single-factor test were acceptable, and a series of procedural measures were implemented during the data collection process, including standardized pre-test instructions, the inclusion of reverse-scored questions, and anonymous questionnaire filling, to reduce such biases, residual biases may still persist. Multi-informant (e.g., teacher) and multi-method approaches (e.g., observational measures) could be used when studying relationships between parental neuroticism and children’s outcomes in the future (Podsakoff et al., 2003). Second, generalizability is constrained by sample composition: 80.4% were mothers, and most participants were urban with high education and income in Guangdong. This limits applicability to father-primary or lower-SES/rural families. Future studies with more balanced and diverse samples are needed to confirm generalizability across parents and family contexts. Third, although this study focused on variables such as parental neuroticism and emotional distress, risk and protective factors for children’s EBPs—particularly those within the family domain—were not fully captured. Future studies should incorporate a broader range of variables to identify additional factors that may affect parents’ emotional distress and children’s EBPs. These may include parent–child relationship, parenting style, marital conflict, parents’ history of mental illness, children’s temperament, sleep, and screen exposure (Prinzie et al., 2005; Tackett, 2006). Additionally, neuroticism was assessed using the 3-item CBF-PI-15 subscale to minimize participant burden. Although this brief measure does not capture all facets of the construct, the three items tap into core features conceptually proximal to emotional distress—namely, worry, emotional reactivity, and stress vulnerability (Costa & McCrae, 1992). These features are central to negative affectivity, which has been consistently linked to parenting and family functioning (Belsky, 1984). We interpret our findings with caution regarding construct breadth and suggest that future research using longer inventories could explore whether specific facets exert differential effects on child outcomes through distinct pathways. Finally, this study cannot provide causal results about relationships because it used a cross-sectional research design. Therefore, more studies need to be conducted with longitudinal data to clarify the direction of causality among parental neuroticism, emotional distress, and children’s EBPs, and to explore whether these patterns persist as the children grow up (Kotov et al., 2010).

## 5. Conclusions

This study applied SEM to examine the interrelationships among parental neuroticism, emotional distress, and their preschool children’s EBPs. More specifically, the most important finding is that parental neuroticism was highly correlated with emotional distress (e.g., anxiety, depression, and somatization) and their preschool children’s EBPs. Parental emotional distress plays a mediating role in the relationship between neuroticism and children’s EBPs. These findings underscore the importance of focusing on the intergenerational association between parental neuroticism and emotional and behavioral development in preschool children.

To achieve a more in-depth understanding of children’s EBPs, we also construct a comprehensive network of emotional and behavioral problems at the symptom level. The network analysis revealed “constantly fidgeting or squirming”, “stealing from home, kindergarten, or other places”, “often unhappy‚ depressed or tearful”, and “many fears‚ easily scared” as the most central symptoms. As such, these may constitute central symptoms for the development and/or maintenance of co-occurring emotional and behavioral difficulties within this group. Timely, multilevel interventions targeting central symptoms may help to alleviate the EBPs in this population. Our findings offer some directions for interventions to lessen EBPs among preschool children and to improve the mental health of preschool children.

## Figures and Tables

**Table 1:** Participant characteristics.

Parents Sample (*N* = 1216)			
Characteristics	Categories	*N*	%
Parental age (years)	Less than 30	83	6.8
	30–39	854	70.2
	40–50	249	20.5
	Missing value	30	2.5
	Total	1216	100
Gender of parents	Fathers	238	19.6
	Mothers	978	80.4
Educational attainment	Primary school education and below	28	2.3
	Junior high school education	83	6.8
	High school education	147	12.1
	College degree	807	66.4
	Master’s degree or higher	151	12.4
Family monthly income	Less than 5000 RMB	119	9.8
	5000 RMB–9999 RMB	349	28.7
	10,000 RMB–20,000 RMB	362	29.8
	Over 20,000 RMB	386	31.7
**Children Sample (*N* = 1216)**			
Children’s age (years)	Total	1216	100
Class of preschool-aged children	Junior class of kindergarten	368	30.3
	Middle class of kindergarten	496	40.8
	Senior class of kindergarten	352	28.9
Gender of children	Girls	579	47.6
	Boys	637	52.4
EBPs			
difficulty level of EBPs	Abnormal	122	10%
	Borderline	121	10%
	Normal	973	80%

Note. EBPs = emotional and behavioral problems.

**Table 2:** Measurement model: factor loadings of latent variables.

Variable	Standardized Factor Loading	Standard Error	Unstandardized Factor Loading	Mean	Standard Deviation
Neuroticism					
Neuroticism1	0.678	0.025	1.000	2.97	1.34
Neuroticism2	0.888	0.014	1.300	2.85	1.33
Neuroticism3	0.892	0.014	1.333	2.70	1.36
Emotional Distress					
Depression	0.919	0.014	1.352	7.58	3.05
Anxiety	0.958	0.007	1.423	7.54	3.08
Somatization	0.853	0.022	1.000	7.06	2.43
EBPs					
Emotional symptoms	0.758	0.029	1.000	1.56	1.71
Conduct problems	0.647	0.032	0.653	2.10	1.31
Hyperactive-inattention	0.535	0.027	0.659	3.45	1.98
Peer relationships	0.496	0.027	0.759	2.28	1.60

## Data Availability

The data presented in this study are available on request from the corresponding author due to privacy restrictions.

## Supplementary Materials

The following supporting information can be downloaded at: https://www.mdpi.com/article/10.3390/bs16071135/s1, Figure S1: Accuracy of edge weights; Figure S2: Stability of node centrality indices and edges; Figure S3: Bootstrapped difference test of edge weights and expected influence; Table S1: Edge weight matrix of EBPs symptoms network.

## Abbreviations

The following abbreviations are used in this manuscript:

EBPs	Emotional and Behavioral Problems
SEM	Structural Equation Modeling
CFA	Confirmatory Factor Analysis
