# Supplementary material for: The Mediating Role of Parental Emotional Distress in the Relationship Between Neuroticism and Children’s Emotional and Behavioral Problems: A Network Analysis and Structural Equation Modeling Study
Source: Behav Sci (Basel). 2026 Jul 6;16(7):1135. doi: 10.3390/bs16071135 (PMC13405562; doi:10.3390/bs16071135)

Table S1.

Edge weight matrix of EBPs symptoms network.

	SDQ3	SDQ8	SDQ13	SDQ16	SDQ24	SDQ5	SDQ7	SDQ12	SDQ18	SDQ22	SDQ2	SDQ10	SDQ15	SDQ21	SDQ25	SDQ6	SDQ11	SDQ14	SDQ19	SDQ23
**SDQ3**	0.000	0.076	0.041	0.010	0.098	0.000	-0.002	0.069	0.049	0.094	0.000	0.080	0.000	0.000	0.000	0.015	0.000	0.000	0.036	0.008
**SDQ8**	0.076	0.000	0.146	0.031	0.105	0.000	-0.042	0.078	0.064	0.114	0.000	0.131	0.000	0.000	-0.041	0.139	0.000	0.000	0.042	0.041
**SDQ13**	0.041	0.146	0.000	0.059	0.062	0.212	0.000	0.095	0.076	0.104	0.000	0.112	0.000	0.000	0.000	0.080	0.000	0.000	0.022	0.000
**SDQ16**	0.010	0.031	0.059	0.000	0.253	0.066	0.000	0.000	0.000	0.000	0.000	0.000	0.061	0.051	0.000	0.080	0.000	0.000	0.050	0.000
**SDQ24**	0.098	0.105	0.062	0.253	0.000	0.027	-0.084	0.000	0.000	0.014	0.000	0.063	0.058	0.000	0.000	0.096	0.017	0.034	0.109	0.069
**SDQ5**	0.000	0.000	0.212	0.066	0.027	0.000	0.038	0.064	0.000	0.000	0.154	0.085	0.054	0.039	0.000	0.018	0.000	0.000	0.010	0.000
**SDQ7**	-0.002	-0.042	0.000	0.000	-0.084	0.038	0.000	0.000	0.000	-0.043	0.000	0.000	-0.014	0.149	0.037	-0.033	0.000	0.060	-0.040	-0.028
**SDQ12**	0.069	0.078	0.095	0.000	0.000	0.064	0.000	0.000	0.189	0.230	0.061	0.072	0.000	0.000	0.000	0.006	0.014	0.031	0.029	0.000
**SDQ18**	0.049	0.064	0.076	0.000	0.000	0.000	0.000	0.189	0.000	0.223	0.000	0.076	0.068	0.000	0.000	0.009	0.000	0.000	0.130	0.005
**SDQ22**	0.094	0.114	0.104	0.000	0.014	0.000	-0.043	0.230	0.223	0.000	0.000	0.111	0.000	-0.039	-0.023	0.055	0.084	0.000	0.067	0.020
**SDQ2**	0.000	0.000	0.000	0.000	0.000	0.154	0.000	0.061	0.000	0.000	0.000	0.139	0.321	0.079	0.094	0.000	0.000	0.000	0.014	0.022
**SDQ10**	0.080	0.131	0.112	0.000	0.063	0.085	0.000	0.072	0.076	0.111	0.139	0.000	0.075	0.000	0.000	0.010	0.024	0.028	0.039	0.000
**SDQ15**	0.000	0.000	0.000	0.061	0.058	0.054	-0.014	0.000	0.068	0.000	0.321	0.075	0.000	0.010	0.229	0.000	0.000	0.000	0.015	0.007
**SDQ21**	0.000	0.000	0.000	0.051	0.000	0.039	0.149	0.000	0.000	-0.039	0.079	0.000	0.010	0.000	0.327	0.000	0.007	0.120	0.000	-0.043
**SDQ25**	0.000	-0.041	0.000	0.000	0.000	0.000	0.037	0.000	0.000	-0.023	0.094	0.000	0.229	0.327	0.000	0.000	0.028	0.119	0.000	-0.063
**SDQ6**	0.015	0.139	0.080	0.080	0.096	0.018	-0.033	0.006	0.009	0.055	0.000	0.010	0.000	0.000	0.000	0.000	0.008	0.036	0.119	0.006
**SDQ11**	0.000	0.000	0.000	0.000	0.017	0.000	0.000	0.014	0.000	0.084	0.000	0.024	0.000	0.007	0.028	0.008	0.000	0.283	0.000	0.000
**SDQ14**	0.000	0.000	0.000	0.000	0.034	0.000	0.060	0.031	0.000	0.000	0.000	0.028	0.000	0.120	0.119	0.036	0.283	0.000	0.000	0.000
**SDQ19**	0.036	0.042	0.022	0.050	0.109	0.010	-0.040	0.029	0.130	0.067	0.014	0.039	0.015	0.000	0.000	0.119	0.000	0.000	0.000	0.000
**SDQ23**	0.008	0.041	0.000	0.000	0.069	0.000	-0.028	0.000	0.005	0.020	0.022	0.000	0.007	-0.043	-0.063	0.006	0.000	0.000	0.000	0.000

Figure S1. ***Accuracy of edge weights.*** This is nonparametric bootstrap tests of edge weights of the EBPs symptoms network. The shades represent the 95% confidence interval for the indices.

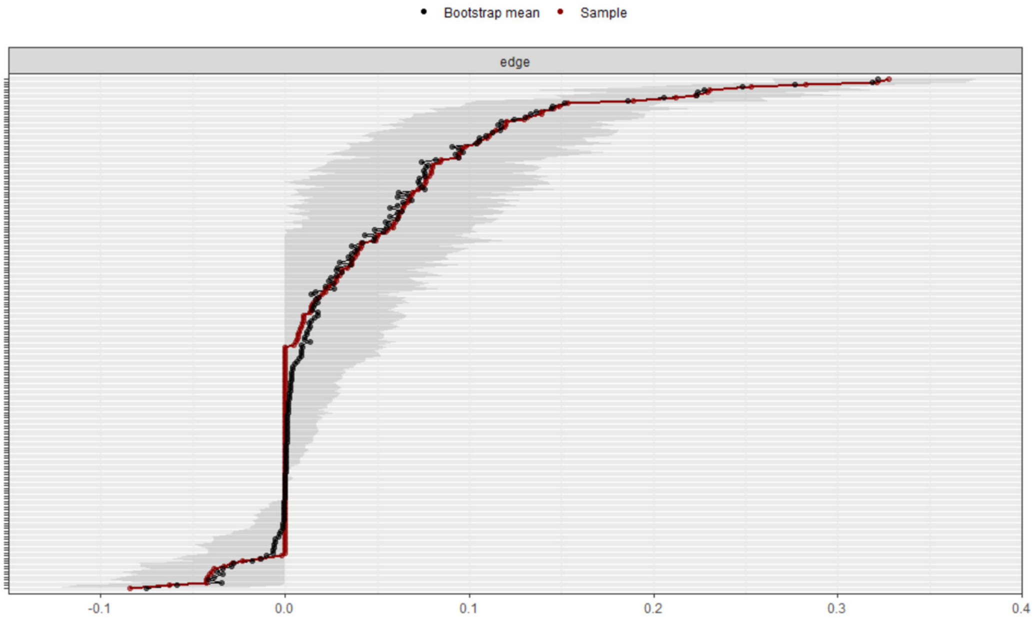

Figure S2. ***Stability of node centrality indices and edges.***
This shows the stability of node centrality indices (strength and expected influence) for the EBPs symptom network. The shades represent the 95% confidence interval for the indices.

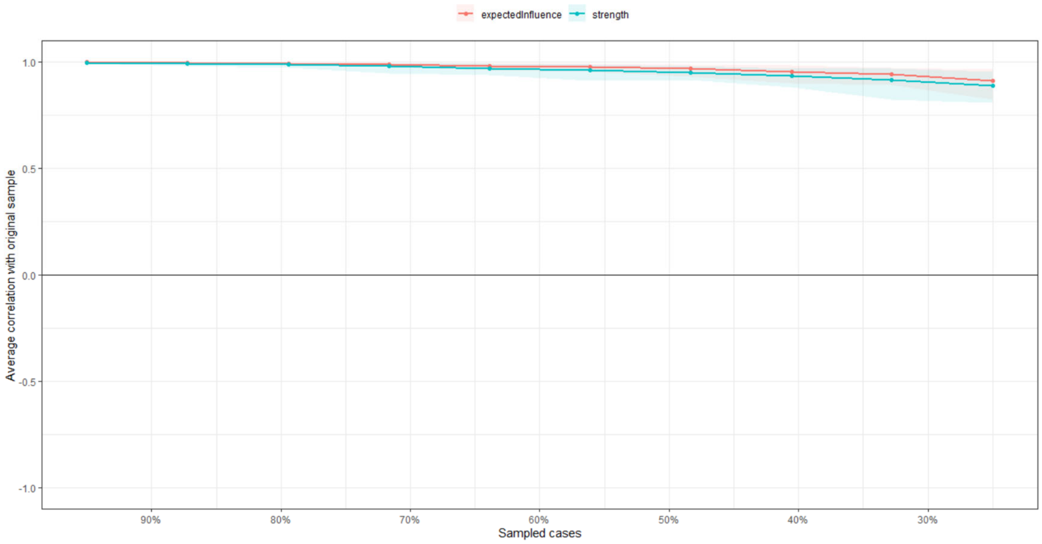

Figure S3. ***Bootstrapped difference test of edge weights and expected influence.*** **(a)** and (**b)** show the difference tests of edge weights and expected influence, respectively, for the EBPs network. Dark squares indicate that the edge weights or the expected influence of the corresponding nodes differ significantly from one another (a = 0.05). Grey squares mean that the edge weights or the expected influence of the corresponding nodes don’t significantly differ from one another. In the plots of the edge weight difference test, blue boxes represent positive correlations while red boxes represent negative correlations.

(a)

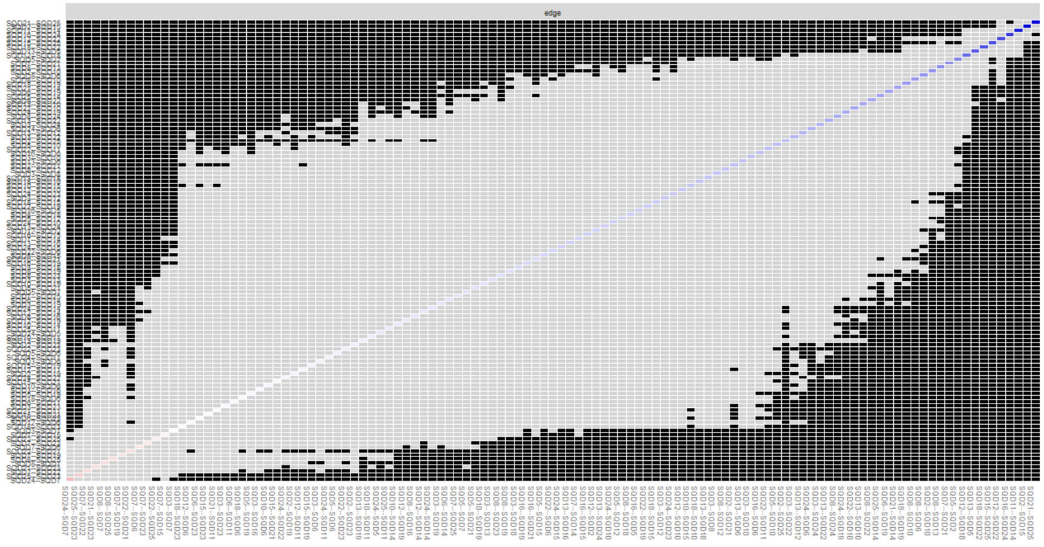

(b)

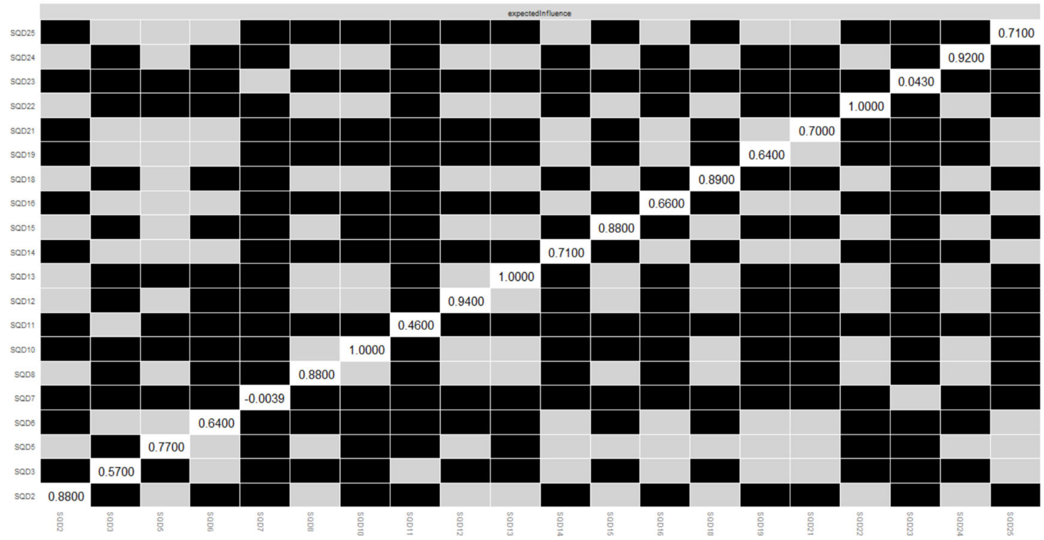

Supplement: Supplementary file 1 [file behavsci-16-01135-s001.zip › behavsci-4361458-supplementary.pdf]
